# A reversal coarse-grained analysis with application to an altered functional circuit in depression

**DOI:** 10.1002/brb3.173

**Published:** 2013-09-22

**Authors:** Shuixia Guo, Yun Yu, Jie Zhang, Jianfeng Feng

**Affiliations:** 1 College of Mathematics and Computer Science, Key Laboratory of High Performance Computing and Stochastic Information Processing (Ministry of Education of China), Hunan Normal UniversityChangsha, Hunan, China; 2 Centre for Computational Systems Biology, School of Mathematical Sciences, Fudan UniversityShanghai, China; 3 Department of Computer Science, University of WarwickCoventry, U.K

**Keywords:** Reversal coarse-grained analysis, source location, voxel-wise time series

## Abstract

**Introduction:**

When studying brain function using functional magnetic resonance imaging (fMRI) data containing tens of thousands of voxels, a coarse-grained approach – dividing the whole brain into regions of interest – is applied frequently to investigate the organization of the functional network on a relatively coarse scale. However, a coarse-grained scheme may average out the fine details over small spatial scales, thus rendering it difficult to identify the exact locations of functional abnormalities.

**Methods:**

A novel and general approach to reverse the coarse-grained approach by locating the exact sources of the functional abnormalities is proposed.

**Results:**

Thirty-nine patients with major depressive disorder (MDD) and 37 matched healthy controls are studied. A circuit comprising the left superior frontal gyrus (SFGdor), right insula (INS), and right putamen (PUT) exhibit the greatest changes between the patients with MDD and controls. A reversal coarse-grained analysis is applied to this circuit to determine the exact location of functional abnormalities.

**Conclusions:**

The voxel-wise time series extracted from the reversal coarse-grained analysis (source) had several advantages over the original coarse-grained approach: (1) presence of a larger and detectable amplitude of fluctuations, which indicates that neuronal activities in the source are more synchronized; (2) identification of more significant differences between patients and controls in terms of the functional connectivity associated with the sources; and (3) marked improvement in performing discrimination tasks. A software package for pattern classification between controls and patients is available in Supporting Information.

## Introduction

The human brain is a complex system with dynamic interactions among various brain regions that operate in a large-scale network. Functional magnetic resonance imaging (fMRI), which has been applied widely in understanding the interworking of the brain, has provided an unprecedented opportunity to study various brain disorders, such as depression, Alzheimer's disease, and schizophrenia, and may represent the key to the early diagnosis of such diseases. When studying the brain using fMRI data, a coarse-grained analysis – dividing the whole brain into regions of interest (ROIs) – is often adopted to investigate the functional connectivity (Ogawa et al. 1990, 1992; Humphreys et al. 2007; Kriegeskorte et al. 2010; Guo et al. 2012) between spatially distributed areas. In this approach, an ROI-wise time series for each region is obtained by simply averaging the time series of all voxels within a specific, coarse brain region (Bassett et al. 2009; Bullmore and Sporns 2009; Dosenbach et al. 2010). Useful information contained in relatively fine components of the brain may be missed using this approach. This is because spatial averaging may blur fine patterns that display significant differences across patient and control groups. A fine-grained approach – dividing the brain into smaller areas – may encounter a technical barrier, that is, very little aberrant functional connectivities can pass a stringent multicomparison correction. Therefore, it is crucial to perform a reversal coarse-grained analysis to obtain more accurate in loci and, therefore, more biologically precise results.

In our previous paper (Tao et al. 2011), a coarse-grained analysis was carried out in 39 patients with major depressive disorder (MDD) and 37 matched controls to investigate the changes in functional connectivity of patients with MDD during a task-free (resting-state) paradigm. A hate circuit comprising the left superior frontal gyrus (SFGdor), right insula (INS), and right putamen (PUT) exhibited the greatest changes between the patients and controls and the changes in these three regions related to the hate circuit have also been reported in the literatures (Husain et al. 1991; Strakowski et al. 1999, 2002; Kimbrell et al. 2002; Anand et al. 2005; Kempton et al. 2011).

Although these three regions exhibited highest significant changes in patients with MDD than in controls, they were too large (from an anatomical point of view) to provide detailed, clinically relevant information. Coarse brain regions obtained from different template such as automated anatomical labeling (AAL) template (Tzourio-Mazoyer et al. 2002) may contain thousands of voxels, and different parts of the same region may have different functions. Locating a relatively fine subregion showing the greatest change should facilitate the analysis of essential differences between patients with MDD and controls and provide more information for clinicians, thereby helping in decision making in their clinical practice.

In the current paper, we proposed a novel and general approach to reverse the coarse-grained approach and to determine the exact location of functional abnormalities. We applied this approach to the hate circuit. The intensity of each voxel was used to locate the subregions of the three structures and to extract voxel-wise time series by averaging the time series of the source voxels located within each region. Our results demonstrated that the voxel-wise time series extracted from the reversal coarse-grained analysis have several advantages: (1) a larger amplitude of fluctuations that are indicative of more synchronized BOLD signals, (2) more significant differences between patients and controls in functional connectivity links, and (3) a greater effect on connectivity and a better performance regarding discrimination accuracy.

## Methods

### Subjects

Thirty-nine MDD patients (23 females, 16 males; mean age 27.99 ± 7.49 years old; mean education 12.00 ± 3.58 years) were recruited from the in- or outpatient department of the Second Xiangya Hospital of Central South University in Changsha, Hunan province, China. The mean illness duration for MDD patients was 27.27 ± 37.82 months, and the mean score on the 17 items version of Hamilton rating scale for depression (HAMD) was 24.97 ± 4.99. Thirty-seven normal controls (14 females, 23 males; mean age 28.22 ± 6.47 years old; mean education 13.32 ± 3.29 years) were also recruited. The two groups have no significant difference in sex (chi-square test, *P* > 0.05), age analyses of variance (ANOVA, *P* > 0.05), and education (two samples *t*-test, *P* > 0.05). The detailed demographics of these two groups are shown in Table 1.

**Table 1:** Subject demographics

	Depression patients *n* = 39	Controls *n* = 37	*P-*value
Age (year)	27.99 ± 7.7	28.22 ± 6.47	0.964
Education (year)	12.00 ± 3.58	13.32 ± 3.29	0.306
Sex (M/F)	23/16	14/23	0.087
Illness duration (year)	2.42 ± 3.26	n.a.	n.a.
HAMD	24.97 ± 5.07	n.a.	n.a.

All patients met the following inclusion criteria: (1) Current MDD attack as assessed by two experienced psychiatrists using the Structural Clinical Interview for Diagnostic and Statistical Manual of Mental Disorders (DSM-IV); (2) 18–45 years of age; (3) right-handed Han Chinese; (4) HAMD scores of at least 17.

Patients and healthy controls were excluded if they had any of the following: (1) a history of neurological diseases or other serious physical diseases; (2) a history of electroconvulsive therapy; (3) history of substance abuse (i.e., drugs, alcohol, and other psychoactive substance); (4) any contraindications for MRI. This study was approved by the Ethics Committee of the Second Xiangya Hospital, Central South University, Hunan, China. Written informed consent was obtained from all subjects.

### Imaging acquisitions and data preprocessing

All image data were acquired using a 1.5T Siemens GE Signa Twinspeed Scanner (General Electric Medical System, Milwaukee, WI). The resting-state fMRI was performed with a gradient-echo echo planar sequence. Subjects were asked to relax and think of nothing in particular with eyes closed but were requested not to fall asleep. A total of 180 volumes of echo-planar imagings were obtained axially (repetition time, 2000 msec; echo time, 40 msec; slices, 20; thickness, 5 mm; gap, 1 mm; field of view (FOV), 24′24 cm^2^; resolution, 64′64; flip angle, 90 degrees).

Prior to preprocessing, the first 10 volumes were discarded to allow for scanner stabilization and the subjects’ adaptation to the environment. fMRI data preprocessing was then conducted by SPM8 (http://www.fil.ion.ucl.ac.uk/spm) and a data processing assistant for resting-state fMRI (DPARSF) (Yan and Zang 2010). Briefly, the remaining functional scans were first corrected for within-scan acquisition time differences between slices, and then realigned to the middle volume to correct for interscan head motions. Subsequently, the functional scans were spatially normalized by using T1 image unified segmentation (done by DPARSF directly) and resampled to 3 × 3×3 mm^3^. After normalization, the BOLD signal of each voxel was first detrended to abandon a linear trend and then passed through a band-pass filter (0.01–0.08 Hz) to reduce low-frequency drift and high-frequency physiological noise. Finally, nuisance covariates including head motion parameters, global mean signals, white matter signals, and cerebrospinal signals were regressed out from the BOLD signals. The masks of three ROIs related to the hate circuit (left SFGdor, right INS, and right PUT) were generated using the free software WFU_PickAtlas (http://www.ansir.wfubmc.edu) (Maldjian et al. 2003). Time series of each voxel within three ROIs were extracted. There were 1184, 597, and 352 voxels, respectively, in these three ROIs.

### Source location

In our previous paper, a coarse-grained analysis, which is conducted by dividing the whole brain into 90 AAL ROIs was performed to investigate the significant changes between patients with MDD and matched controls. A so-called hate circuit composing of left SFGdor, right INS, and right PUT exhibited the greatest changes. Here, a reversal coarse-grained analysis, conducted by dividing the whole brain into voxels, was carried out to these three regions to find out the essential difference between these two groups. First of all, the Pearson correlation coefficient of all interregional links (for voxels) are calculated between SFGdor versus INS and INS versus PUT, a fisher's r-to-z transformation is utilized to convert each correlation coefficient *r*_*ij*_ into *Z*_*ij*_ to improve the normality. After that, a two-sample t-test was then performed for all interregional links. A significance level of *P* < 0.05 was used to find out the dysfunctional links. As pointed in Zhang et al. (2012), the functional connectivity between two voxels is very sensitive to noise which is ineluctable even after filtering, the located source regions are not usually robust. To circumvent this problem, for each selected voxel *i* in region A, a dysfunction intensity for this voxel is defined as following:


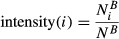


where 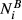 is the number of voxels in region B that show significantly different (*P* < 0.05) functional connectivity with the selected voxel *i* in region A compared with normal controls. *N*^*B*^ is the total number of voxels in region B. This is reasonable as the value of intensity represents the significance of the functional abnormality for each voxel. In the following study, we use 0.15 as threshold of intensity to locate the source regions.

### Statistics for assessing functional link alterations

When studying the functional connectivity, we are interested in knowing not only whether a link between two ROIs is statistically significant, but also the level, or strength, of this alteration. The “measures of effect” are indexes that summarize the strength of the change of a link between two regions, which are more informative in investigating the essential difference between different groups. The strength of the link can be measured both in relative and absolute terms (Wacholder 1986; Bland and Altman 2000; Pepe et al. 2004; Tao et al. 2011), which will be introduced in the following subsection.

#### Odds ratio

The odds ratio is a relative measure of the effect of a certain link, describing the strength of association, or nonindependence, between two binary data sets. After constructing the functional maps for both depression patients and healthy controls, we can compare the effect of a certain link using the odds ratio, which is defined as the ratio of the odds occurring in the patients to the odds occurring in the healthy controls. Here, we use *P*_11_ and *P*_01_ to denote the number of links occurring in the patients’ group and in the healthy controls’ group, respectively, and we use *P*_10_ and *P*_00_ to denote the number of links not occurring in the patients’ group and in the healthy controls’ group, respectively. The odds ratio of this link is


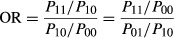


The odds ratio is generally accompanied by a measure of the precision of the estimate: the confidence interval (CI). The 1−α confidence interval of the odds ratio is


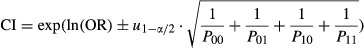


where *u*_1−α/2_ is the 1−α/2 quantile of the standard normal distribution. An odds ratio of 1 indicates that the link is equally likely to occur in both groups. The lower confidence level of an odds ratio greater than 1 indicates that the link is a dangerous factor and is more likely to occur in the patients’ group, and the upper confidence level of an odds ratio less than 1 indicates that the link is a protective factor and is less likely to occur in the patients’ group.

#### Risk difference

The effect associated to a certain link can also be evaluated in terms of absolute risk difference (Tripepi et al. 2007), that is, the difference between the occurrence proportion of a link in the patients’ group and that in the healthy controls’ group. Specifically, the risk difference is defined as follows for a particular link:


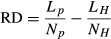


Where *L*_*p*_ and *L*_*N*_ are the number of a certain link presents in the individual network of the patients and the control group, respectively, and *N*_*p*_ and *N*_*N*_ are the total number of patients and the healthy controls. Similarly, a risk difference of 0 indicates that the link is equally likely to occur in both groups, lower confidence level of a risk difference greater than 0 indicates that the link is a dangerous factor and is more likely to occur in the patients’ group, and upper confidence level of a risk difference less than 0 indicates that the link is a protective factor and is less likely to occur in the patients’ group. In order to obtain the statistical significance of risk difference of a certain link, a permutation test can be carried out.

### Amplitude of low-frequency fluctuation analysis

The amplitude of low-frequency fluctuation (ALFF) is calculated for both ROI-wise data and voxel-wise data. In brief, after band-passing filtering (0.01–0.08 Hz) and linear-trend removal, the ROI-wise time series and the voxel-wise time series are extracted within each of the three ROIs, which are then transformed to the frequency domain using a fast Fourier transform to obtain the power spectrum. As the power of a given frequency is proportional to the square of its amplitude in the original time series, the power spectrum obtained by a fast Fourier transform is squared root transformed and then averaged across 0.01–0.08 Hz to yield a measure of ALFF for the ROI-wise time series and the voxel-wise time series, respectively, for three ROIs. Two sample t-test can be carried out to test the significant changes in both the ROI-wise data and voxel-wise data (Yang et al. 2007; Lui et al. 2010).

### SVM classifier

The support vector machine (SVM) is a learning machine for classification problem with two class. As it was first proposed by Vapnik as a logistical extension of statistical learning theory, SVM has become widely used in many areas because of its ability to handle high-dimensional data, and its accuracy in classification and prediction. Because of such properties, it has proven a powerful tool in the analysis of fMRI data.

SVM conceptualizes the idea that vectors are nonlinearly mapped to a very high dimension feature space. In the feature space, a linear separation surface is created to separate the training data by minimizing the margin between the vectors of the two classes. The training ends with the definition of a decision surface that divides the space into two subspaces, each subspace corresponding to one class of the training data. Once the training is completed, the test data are mapped to the feature space. A class is then assigned to the test data depending on which subspace they are mapped to (Hastie et al. 2001; LaConte et al. 2005; Mourão-Miranda et al. 2007).

In this study, a SVM toolkit named libsvm written by Lin Chih-Jen from Taiwan University (http://www.csie.ntu.edu.tw/∼cjlin/libsvm/) is used. Radial basis function (RBF) is selected as kernel function (*t* = 2), parameter C is fixed to 10, which is used to trade-off learning and extend ability, and other parameters are kept as default values.

## Results

### Source location

There are 1184 voxels in left SFGdor, 597 voxels in right INS, and 352 voxels in right PUT. For the SFGdor–INS link, we calculate all interregion correlation coefficients and obtain the intensity of each voxel within the corresponding two ROIs. Similar analysis is performed for the INS–PUT link and the intensity of the voxel within right INS and right PUT is also obtained. Here, we define the intensity of the voxels within INS to be the maximum intensity obtained from SFGdor–INS link and INS–PUT link. An intensity level (intensity >0.15) is used to threshold voxels within these three regions into two groups (unchanged part and changed part). There were 202 voxels with significant changes in the left SFGdor, 188 voxels in the right INS and 84 voxels in the right PUT.

**Figure 1:**
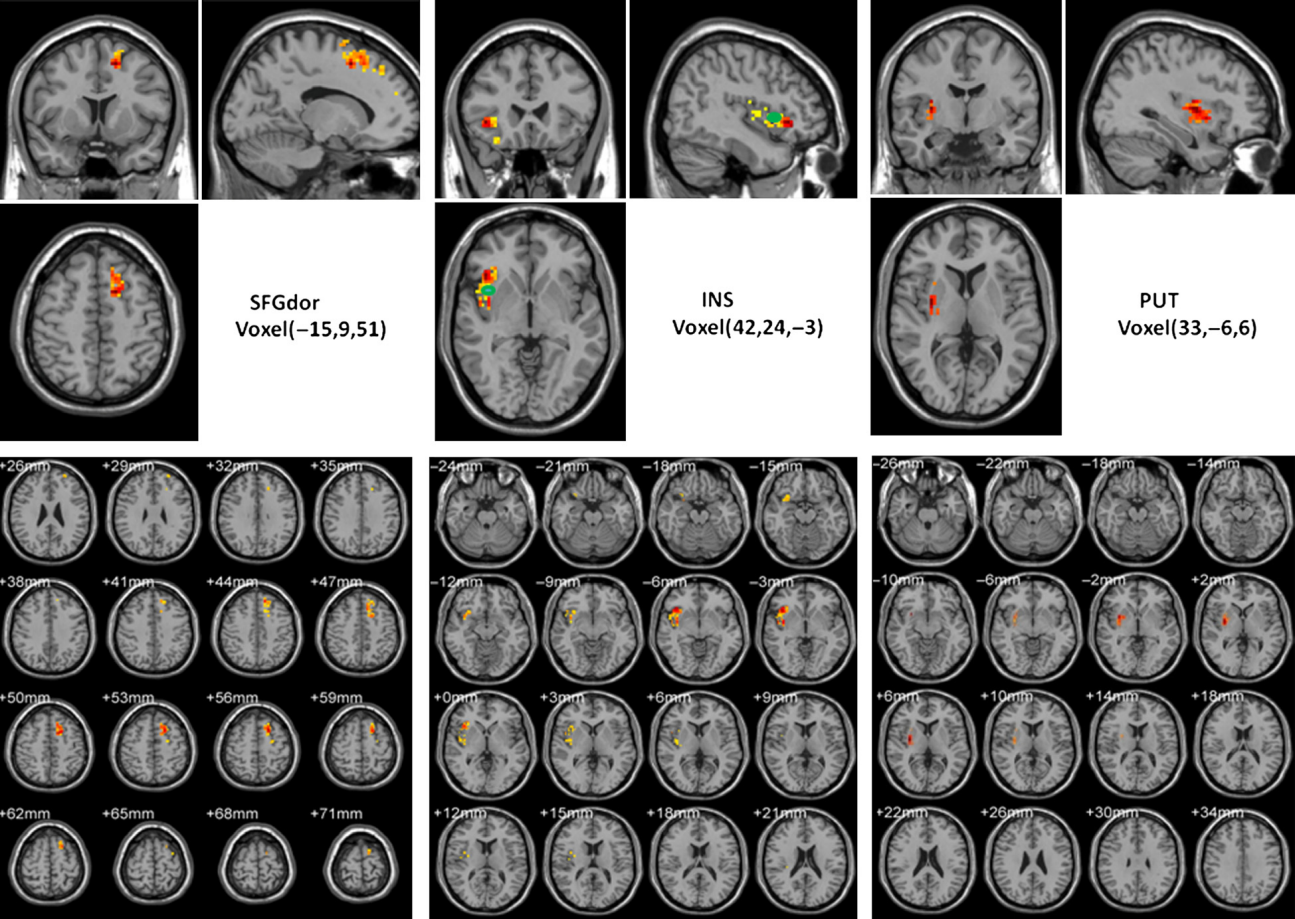
(Upper) Axial, coronal, and sagittal view of source voxels detected from SFGdor (left), INS (middle), and PUT (right). The peak coordinates of source voxels are (−15, 9, 51), (42, 24, −3), and (33, −6, 6), respectively. In the plot of INS, the green color represents the core subregion. (Bottom) Multislices view of the source voxels of SFGdor (left), INS (middle), and PUT (right).

The detected parts within these three regions are plotted as warm colors in Figure 1. The upper three panels are axial, coronal, and sagittal view detected from SFGdor (left), INS (middle), and PUT (right), respectively. The center coordinates representing the Montreal Neurological Institute (MNI) coordinates of the most significantly changed voxels within the regions of SFGdor, INS, and PUT are (−15, 9, 51), (42, 24, −3), and (33, −6, 6), respectively. In the plot of INS, a green color represents the core subregion located from both SFGdor–INS link and INS–PUT link. The bottom panels are multislice view of the source subregion of SFGdor (left), INS (middle), and PUT (right), respectively.

**Figure 2:**
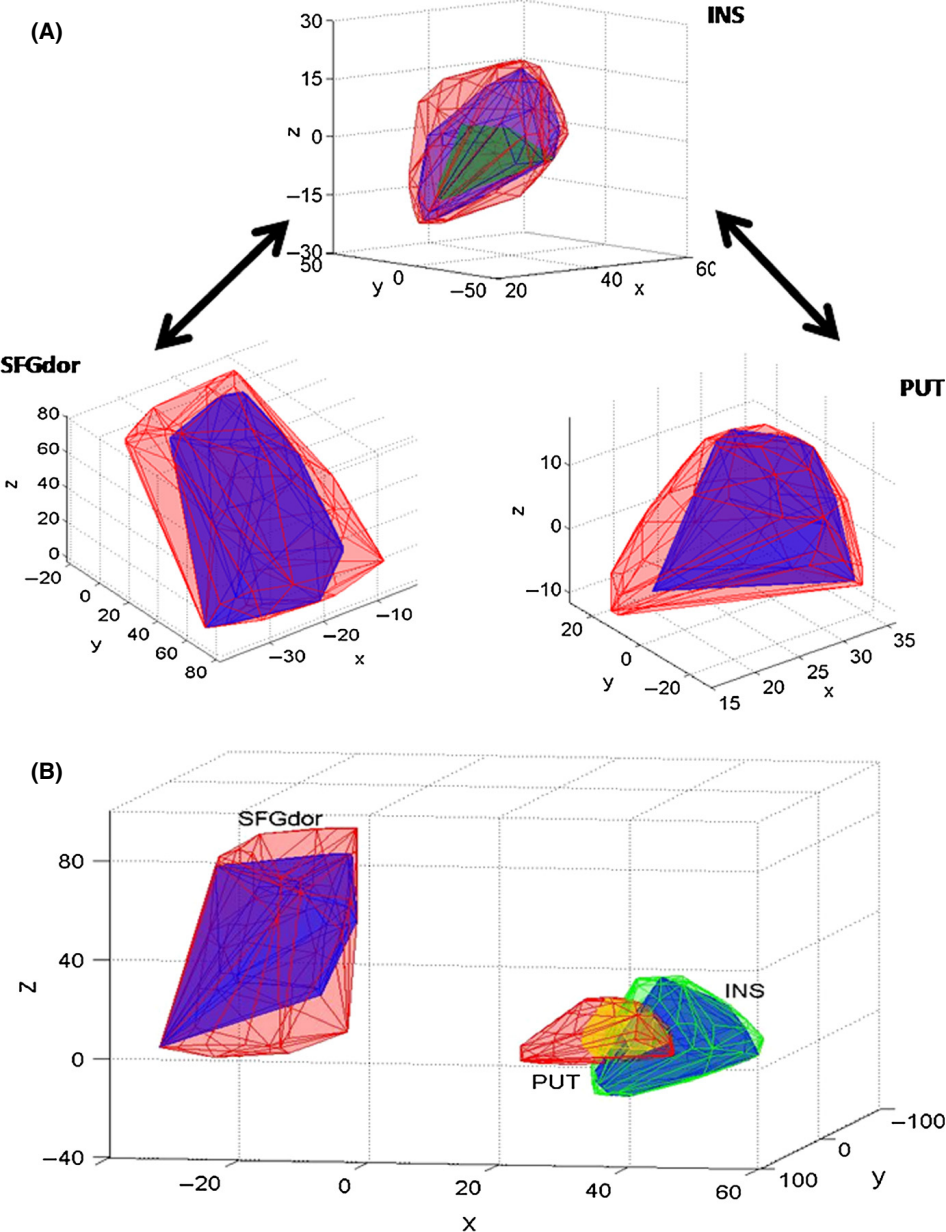
Visualization of the source voxels within the left superior frontal gyrus (SFGdor), the right insula (INS), and the right putamen (PUT). (A) The outer red contour is the original shape of the whole region while the inner blue contour is the shape of the source voxels. There are two links in the hate circuit: SFGdor–INS and INS–PUT which are represented by the two arrows. (B) General visualization of the hate circuit.

Figure 2 is the visualization of the source subregion within three ROIs related to the hate circuit. In Figure 2A, the outer red contour represents the shape of the original whole area while the inner blue contour denotes the shape of the source subregions. In the plot of INS, the green contour is the shape of the core subregion. It is clear that the source the subregions obtained within all these three ROIs by our reversal coarse-grained approach is more concentrated, lying almost in the middle part of the whole region. The two links in the hate circuit: SFGdor–INS and INS–PUT are represented by two arrows. Figure 2B is the general visualization of the hate circuit. The video of this visualization can be found in Supporting Information.

### Clustering properties

After source location in the three ROIs (SFGdor, INS, PUT), the next step is to calculate the MNI coordinates of the source voxels and identify the peak MNI coordinate of the most significantly changed voxel within each ROI. Furthermore, in order to extract features from the located source voxels within each ROI, we also need to evaluate the clustering properties of these voxels. Here, we use the command BWLABELN in matlab to directly compute connected components for binary images and use a three-dimensional 18-connected neighborhood as the connectivity criterion to obtain the number and the size of clusters (Zou 2004). The results are listed in Table 2.

**Table 2:** Demographic of the source voxels of hate circuit

ROI	SFGdor	INS	PUT
Number of source voxels	202	188	84
Largest cluster (percentage)	17%	31%	25%
Number of clusters	8	9	3
Size of the largest cluster	164	178	81
Coordinate of the peak	(−15, 9, 51)	(42, 24, −3)	(33, −6, 6)
Peak intensity	0.53601	0.55398	0.34171
*P-*value	0.0175	0.0196	0.0397

For left superior gyrus, there are 202 source voxels found in total, accounting for 17% of the whole region. By using BWLABELN in matlab directly to the binary image, we obtain eight clusters. The largest cluster has 164 voxels with peak MNI coordinate being (−15, 9, 51) and peak intensity is 0.536 with *P* = 0.0175. The second largest cluster has 14 voxels and third largest cluster has 7 voxels and the fourth cluster have 4 voxels and the remaining one cluster has only 1 voxel.

In the right INS, there are altogether 188 source voxels, which account for 31% of the whole region. We obtain nine clusters with the largest one having 178 voxels, peak MNI coordinate being (42, 24, −3), and peak intensity is 0.55,398 with *P* = 0.0197. The next two clusters have 2 voxels and the remaining six clusters have 1 voxel. The core of the subregion includes 11 voxels, all of which are located in the largest cluster.

In the right PUT, there are 84 source voxels in total, account for 25% of the whole region. We obtain three clusters with the largest having 81 voxels, peak MNI coordinate being (33, −6, 6), and peak intensity is 0.3417 with *P* = 0.0397. The remaining one cluster has 2 voxels with peak MNI coordinate being (27, −15, 6) and one cluster contains 1 voxel with MNI coordinate being (27, 18, −6).

### Comparisons on various statistics

**Figure 3:**
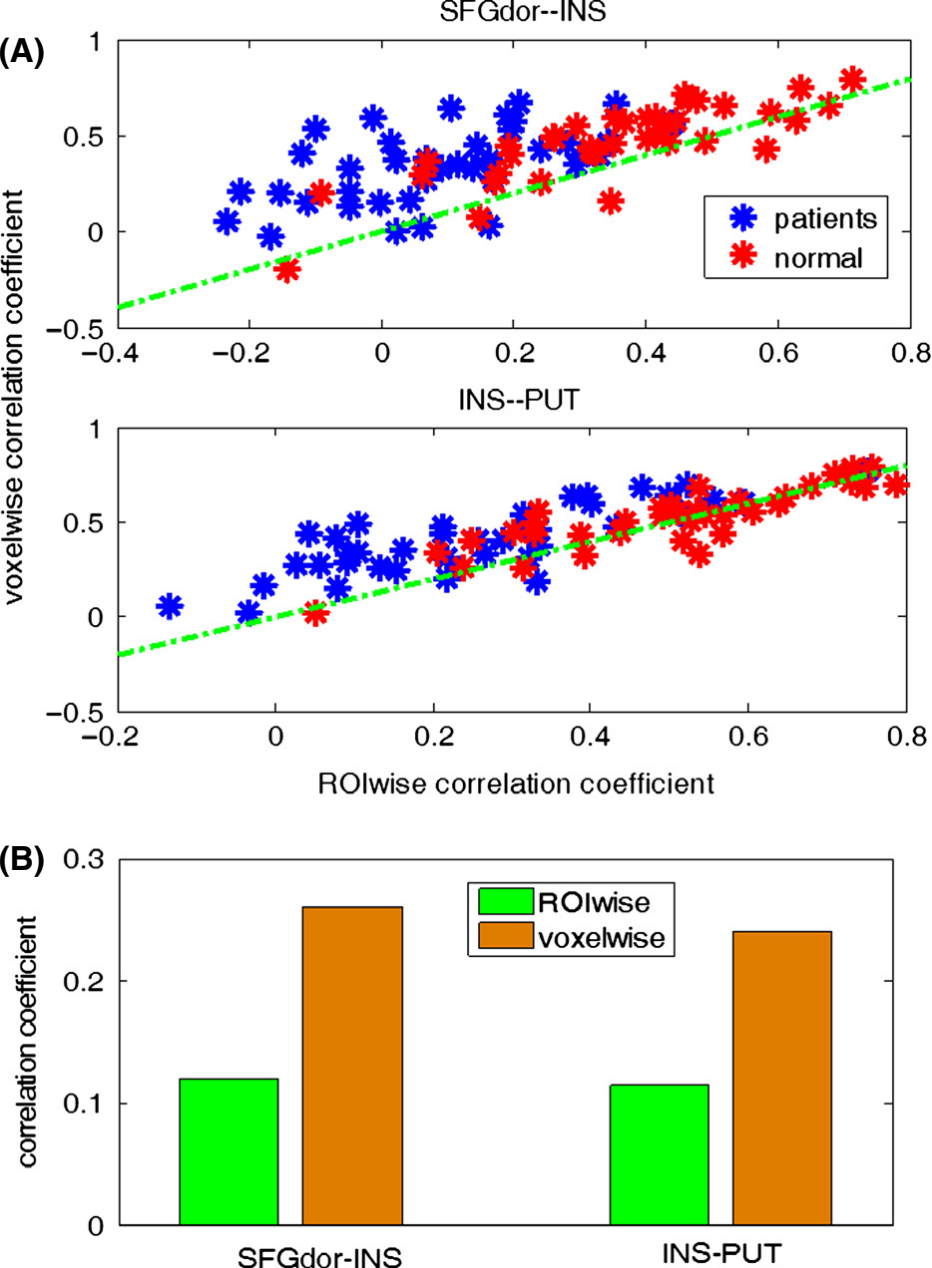
(A) Plot of the correlation coefficient of ROI-wise data and voxel-wise data for SFGdor–INS link and INS–PUT link. (B) Difference of mean correlation coefficients between normal controls and patients. It is clear that the difference of the voxel-wise data is much greater than the ROI-wise data for both links, which means more significant changes can be observed from the voxel-wise data.

After locating source subregions of the hate circuit, the voxel-wise time series, that is, the averaged time series over the identified source, voxels within each ROI can be obtained for further analysis. To compare the ROI-wise time series and the voxel-wise time series in analyzing the two links SFGdor–INS and INS–PUT, for each subject, we plot the correlation coefficients of the two functional connectivity (SFGdor–INS and INS–PUT) using the ROI-wise data and the voxel-wise data, respectively (see Fig. 3A). It can be seen that the correlation coefficients of these two links calculated using ROI-wise data are slightly higher than those obtained using the voxel-wise data both for patients and controls. This observation indicates the reduction of the coherence of activity among the three source subregions. Figure 3B plots the difference of mean correlation coefficients (DOC) between the normal controls and the patients. It is clear that the difference obtained from voxel-wise data is much larger than that from ROI-wise data for both links, which indicates that more significant changes can be detected using the voxel-wise data.

Now we analyze the effect of these two links using both ROI-wise data and voxel-wise data, with the results being listed in Table 3. For SFGdor–INS link, the ROI-wise data have an odds ratio of 0.1270 with *P* = 0.0598, while voxel-wise data have an odds ratio of 0.1484 with *P* = 0.0009, which is far more significant than that from ROI-wise data. For the absolute measure of effect, the ROI-wise data have a risk difference of −0.1525 with *P* = 0.03 (permutation test), while voxel-wise data have a much greater risk difference of −0.3777 with *P-*value being almost zero. Similarly, for INS–PUT link, the ROI-wise data have an odds ratio of 0.5139 with *P* = 0.5934, which is no longer significant. But for voxel-wise data, the corresponding odds ratio is 0.0556 with *P* = 0.0069, which is significant. For the absolute measure of effect, the ROI-wise data have a risk difference of −0.0243 with *P =* 0.6180 (permutation test), while voxel-wise data have a much greater risk difference of −0.3063 with *P* approaching zero.

**Table 3:** Different measures of effect on SFGdor–INS link and INS–PUT link with ROI-wise and voxel-wise data

	SFGdor–INS		INS–PUT	
		
	ROI-wise	Voxel-wise	ROI-wise	Voxel-wise
OR	0.1270	0.1484	0.5139	0.0556
*P-*value	0.0598	0.0009	0.5934	0.0069
RD	−0.1525	−0.3777	−0.0243	−0.3063
*P-*value	0.03	0	0.6180	0
DOC	0.1199	0.2604	0.1143	0.2405

OR, odds ratio; RD, risk difference; DOC, difference of coefficient.

### ALFF

**Figure 4:**
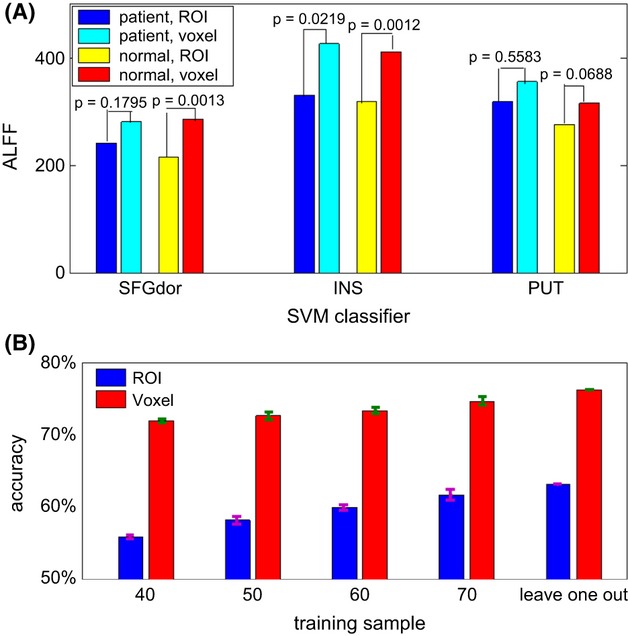
(A) ALFF of the ROI-wise data and the voxel-wise data in SFGdor, INS, and PUT. It is easy to see that ALFF of the voxel-wise data is larger than that of the ROI-wise data both for patients and for normal controls among all these three regions. ALFF have significant difference in SFGdor with normal, in the INS with both patients and normal. (B) Discrimination accuracy of ROI-wise correlation and voxel-wise correlation. It is easy to see that the voxel-wise correlation is helpful for improving discrimination accuracy.

After band-pass filtering (0.01–0.08 Hz) and linear-trend removal, the ROI-wise time series obtained from coarse-grained analysis and the voxel-wise time series obtained from reversal coarse-grained analysis can be extracted. Both of them are transformed to the frequency domain using a fast Fourier transform and the ALFF can thus be obtained. Figure 4A is the ALFF of the ROI-wise data and the voxel-wise data in SFGdor, INS, and PUT. From Figure 4A, it is obvious that the power spectrum of the voxel-wise time series is larger than that of the ROI-wise data for both patients and normal controls among all these three regions, which indicates increased synchronized neuronal connectivity in located subregions (Fox and Raichle 2007; Lui et al. 2010). From Figure 4A, it can be seen that ALFF of the ROI-wise data and the voxel-wise data demonstrate significant differences in the left SFGdor with normal, in the right INS with both patients and normal.

### Predictive power of connectivity changes

In this study, SVM is used to discriminate between subjects belonging to two different classes (i.e., patients and controls). For different training samples, we first select the correlation coefficients from the ROI-wise data of the two links (i.e., SFGdor–INS, INS–PUT) as features to train the model and repeat 5000 times. The trained SVM is then applied to the remaining test data and a mean rate of correct classification for the test data is obtained. It can be seen from Table 4 that the best classification accuracy is 63.96% with a leave-one-out training sample.

**Table 4:** Classification results using ROI-wise and voxel-wise links of the hate circuit

Training sample	40%	50%	60%	70%	Leave one out (%)
ROI-wise					
Accuracy	56.61	58.92	60.5	61.91	63.96
Specificity	50.43	52.94	56.18	58.89	62.55
Sensitivity	65.93	68.11	68.17	67.52	69.05
Voxel-wise					
Accuracy	73.51	74.46	74.95	76.82	77.96
Specificity	66.89	69.36	70.47	72.66	73.67
Sensitivity	83.91	83.84	84.02	86.53	82.97

Next, we perform the discrimination task using the voxel-wise data and compare the results with those from ROI-wise data. For different training samples, we first locate the source voxels in these three regions and select those voxels with intensity level greater than 0.1. Then we extract the voxel-wise time series by averaging the time series of the selected source voxels within each ROI. Again we use the correlation coefficients of the two links (i.e., SFGdor–INS, INS–PUT) with the voxel-wise data as features to train the model and repeat it 5000 times. The trained SVM is then applied to the remaining test data and a mean rate of correct classification for the test data is obtained. From Table 4, we can see that the best classification accuracy is increased to 77.96% with a 14% enhancement of accuracy being obtained. Figure 4B is the bar plot of the discrimination accuracy with a different percentage of training samples. It is easy to see that the voxel-wise data is helpful for improving discrimination accuracy.

## Discussion

Coarse-grained analysis is a general approach that is used to study the functional connectivity of fMRI data by dividing the brain into coarse components (ROIs). Using coarse-grained analysis, one can detect the ROIs or the functional connectivity with significant differences between two groups (Ogawa et al. 1990, 1992; Bassett et al. 2009; Bullmore and Sporns 2009). Previously, the AAL used by us, similar to those reported in many other published papers, showed that one ROI may contain a few thousand voxels and the functional meaning of each ROI is very complex or is a mixture of different functions. Coarse-grained analysis may not provide clear information over these fine spatial scales. Therefore, to identify the essential differences between two groups and specify the biological function for each ROI, we moved a step forward and performed a reversal coarse-grained analysis that would be more informative for disease diagnosis.

In the current paper, a reversal coarse-grained analysis was performed in patients with MDD and matched healthy controls to determine the exact location of the changed site of the functional network described in our previous study. Subregions with the greatest changes were located within three ROIs, that is, left SFGdor, right INS, and right PUT. Previous work has shown that the default mode of network in patients with MDD had undergone significant changes (Greicius et al. 2003; Sheline et al. 2009) in the subcortical area (Goldapple et al. 2004; Zhang et al. 2008; Anand et al. 2009), INS (Liu et al. 2010), and PUT (Husain et al. 1991; Strakowski et al. 1999, 2002). In our current research, although reversal coarse-grained analyses focused specifically on the regions related to the hate circuit, the approach could be easily applied to other circuits or dysfunctional regions.

Here, we proposed a holistic method to locate the source regions by computing the intensity of each voxel. This is logical because the value of intensity represents the significance of alteration in the functional connectivity for each voxel. The measure of intensity is superior to merely thresholding the intervoxel correlation coefficients by *P-*values, as the functional connectivity of two voxels is very sensitive to noise which is ineluctable in our fMRI signal (Friman et al. 2003; Polyn et al. 2005). Another approach to select source voxels was based on the level of information about the patterns of activity expressed over all possible sets of voxels (Norman et al. 2006). Because of the combinatorial explosion issue caused by the large number of possible voxel sets, this approach can be improved further in different ways. Kriegeskorte et al. (2006) proposed scanning the image volume using a “searchlight” and limiting the search to sets of spatially adjacent voxels. All spherical searchlights were assumed to become active as a unit. Different region sizes (the radius of the spherical “searchlight”) were first checked to yield the optimal performance of the “searchlight.” The “searchlight” was then obtained by computing the multivariate effect statistic at each location. Pessoa and Padmala (2007) used an iterative procedure by deleting 1 voxel from the set each time to maximize the goodness of the current voxel set. Their basic idea was to start with all voxels and iteratively eliminate the “least informative” voxel (i.e., the smallest weight) at each cycle. This process was repeated until there was no need to delete voxels. Obviously, this method is more time consuming compared with our approach.

Our results demonstrated that the voxel-wise time series extracted from reversal coarse-grained analysis have a few advantages over ROI-wise time series extracted from coarse-grained analysis. First, the voxel-wise time series had larger ALFFs for all three areas related to the hate circuit (Fig. 4A). In previous studies, ALFF was thought to reflect spontaneous neural activity (Fox and Raichle 2007), which was correlated with activity in gamma-band power (Shmuel and Leopold 2008); this in turn reflects increased regionally synchronized neuronal connectivity and is associated with a capacity for higher cognitive functions (Lewis et al. 2008). The enhancement of ALFF reflects that the regional neuronal populations function in a more synchronous manner within the source subregions than in the entire region. Second, a reversal coarse-grained analysis resulted in the attenuation of functional connectivity, both for the SFGdor–INS and INS–PUT connectivities (Fig. 3A), probably because the localized subregions led to a reduction of the integration of synchronous activity across brain regions. The greater difference of these two links between the two groups indicated that a more significant difference could be detected. However, it is unclear whether this reduced connectivity is beneficial. The pattern of increased ALFF, together with reduced network level connectivity, suggests that increased spontaneous regional neural activity is associated with a parallel reduction, rather than with an enhancement in the coherence of activity across the three source regions (SFGdor, INS, and PUT). Third, reversal coarse-grained analysis had a greater effect (in terms of both odds ratio and risk difference) on functional connectivity (SFGdor–INS and INS–PUT); this means that the identified subregions exhibited significant differences between the two groups.

Our discrimination results showed that feature selection, based on reversal coarse-grained analysis, yields a better classification performance. In fact, many discrimination analyses performed at the voxel level yielded high discrimination accuracy. For example, a classification method was proposed to distinguish subjects of two groups using multiple independent components and their combinations, with the independent component extracted on the basis of the time series of each voxel (Calhoun et al. 2008; Demirci et al. 2008). Although different independent components had different classification performances, the selection of independent components relied on a priori knowledge, and no systematic component selection method was available. Another frequently used discrimination approach is multivoxel pattern analysis (MVPA), which uses pattern-classification techniques to extract the signal across multiple voxels. Many studies have used MVPA to discriminate cognitive changes successfully. For example, MVPA has been used to predict the time course of recall behavior in a free-recall task (Polyn et al. 2005), and it has also been used to predict second-by-second changes in perceived stimulus dominance during a binocular rivalry task (Haynes and Rees 2005). The most important obstacle to the extensive use of the voxel-based discrimination approach is the large number of voxel sets to be scanned. However, if improvements are made in the computational algorithms, the voxel-based approach will be highly promising as a tool for characterizing and understanding of how information is represented and processed in the brain.

In functional connectivity analysis, the term *ROI*-wise or voxel-wise is occasionally used in different documents or software (e.g., in Resting-State fMRI Data Analysis Toolkit (REST) provided by Beijing Normal University (http://www.restfmri.net/), one can calculate ROI-wise or voxel-wise functional connectivity directly), indicating that both ROI-wise analysis and voxel-wise analysis in functional connectivity are seed-based approaches. The ROI-wise analysis estimates the brain connectivity by computing correlation between temporal signals from two predefined ROIs, whereas the voxel-wise analysis correlates functional temporal signals of a seed region with those of other brain voxels (Craddock et al. 2011; Valsasina et al. 2011). The selection of ROIs typically requires a priori knowledge about the underlying problem; therefore, both of these approaches are conceptually different from the reversal coarse-grained method proposed here.

In summary, the current study compared coarse-grained analysis with reversal coarse-grained analysis by analyzing the functional abnormalities of the hate circuit studied previously by us in patients with MDD over a fine spatial scale (Tao et al. 2011). By computing the intensity of each voxel, we were able to precisely localize the changed site of the hate circuit. Furthermore, our results demonstrated that the voxel-wise time series extracted from the reversal coarse-grained analysis had several advantages: (1) a larger amplitude of fluctuations was detected, which indicates that the BOLD signals are more synchronized; (2) more significant differences were observed in the functional connectivity related to the ROIs between patients and controls; and (3) a better performance was observed in the discrimination tasks. From a global perspective, coarse-grained analysis is an appropriate method to investigate the significantly different ROIs and functional connectivity. However, to determine the essential, clinically relevant difference between patients and healthy controls, a reversal coarse-grained analysis should be carried out to identify functional and, possibly, anatomical abnormalities in greater detail.

## Supporting Information

**Data S1.** INS_all.xlsx: coordinate of all INS voxels.

**Data S2.** INS_small.xlsx: coordinate of source INS voxel.

**Data S3.** PUT_all xlsx: coordinate of all PUT voxels.

**Data S4.** PUT_small.xlsx: coordinate of sourcel PUT voxels.

**Data S5.** SFGdor_all. xlsx: coordinate of all SFGdor voxels.

**Data S6.** SFGdor_small.xlsx: coordinate of source SFGdor voxels.

**Data S7.** Myrotate: code for Figure 2.

**Data S8.** Video: code for Figure 2.

**Data S9.** Code for brain-wise association study (BWAS)

**Data S10.** Code for brain-wise association study (BWAS).

**Data S11.** Code for brain-wise association study (BWAS).

**Data S12.** Code for brain-wise association study (BWAS).

**Data S13.** Code for brain-wise association study (BWAS).

**Data S14.** Code for brain-wise association study (BWAS).

**Data S15.** Code for brain-wise association study (BWAS).

**Data S16.** Code for brain-wise association study (BWAS).

**Data S17.** Code for brain-wise association study (BWAS).

**Data S18.** Code for brain-wise association study (BWAS).

**Data S19.** Code for brain-wise association study (BWAS).

**Data S20.** Code for brain-wise association study (BWAS).

**Data S21.** Code for brain-wise association study (BWAS).
